# *Begonia medogensis*, a new species of Begoniaceae from Western China and Northern Myanmar

**DOI:** 10.3897/phytokeys.103.25392

**Published:** 2018-07-02

**Authors:** Jian-Wu Li, Yun-Hong Tan, Xi-Long Wang, Cheng-Wang Wang, Xiao-Hua Jin

**Affiliations:** 1 Center for Integrative Conservation, Xishuangbanna Tropical Botanical Garden, Chinese Academy of Sciences, Menglun, Mengla, Yunnan 666303, P.R. China; 2 South Asia Biodiversity Research Institute, Chinese Academy of Sciences, Yezin, Nay Pyi Taw 05282, Myanmar; 3 Tibet Plateau Institute of Biology, Lhasa 850001, P.R. China; 4 School of Life Sciences, Nanchang University, Nanchang 330031, Jiangxi, P.R. China; 5 Institute of Botany, Chinese Academy of Sciences, Beijing 100093, P.R. China

**Keywords:** *Begonia*, *Begonia medogensis*, sect. *Platycentrum*, new species, China, Myanmar

## Abstract

*Begonia medogensis* JianW.Li, Y.H.Tan & X.H.Jin, a new species of Begoniaceae, is described and illustrated by colour photographs. *Begonia medogensis* is distributed in western China and northern Myanmar. It has erect stems, is tuberless, has many triangular to lanceolate leaves, base slightly asymmetric, margins remotely and irregularly denticulate; staminate flowers have 4 perianth segments, with outer 2 segments broadly ovate, inner 2 spathulate; pistillate flowers have 5 perianth segments, unequal, outer 4 broadly ovate, inner 1 spathulate. The new species is assigned to section Platycentrum and can easily be distinguished from the other species in the section.

## Introduction

*Begonia* L. (1753) is amongst the largest genera in the angiosperms, with more than 1800 species widely distributed in the tropical and subtropical areas of the world (Ku et al. 2007; Chen et al. 2018) and numerous hybrids and cultivars popular in the horticultural market (Gregório et al. 2015). South America and Asia have the richest diversity of *Begonia*, with many new species still being described (such as Chen et al. 2018a, Camfield and Hughes 2018).

During our botanical survey to Medog County, Tibet, western China in late 2017, *Begonia* specimens, including DNA samples, were collected. The DNA samples were kept in a freezer and specimens were deposited in HITBC and PE for further study. The same species has been discovered in our botanical survey of Kachin State, northern Myanmar, in 2017. Results from our study indicate that it is a species new to science, which we describe here.

This new species belongs to section Platycentrum (Klotzsch) A. DC., characterised by terrestrial plants, tubers usually absent, rhizomatous or with upright stems, leaves more than 2, not peltate, usually simple, flower usually without bracteoles, male flower with 4 (rarely 2) free perianth segments, female flower with 4–6 (rarely 3 or 8) free perianth segments, ovary with 3 very unequal wings, locules 2. Besides this new species, there are 171 other species of Begonia section Platycentrum (Klotzsch) A.DC. distributed in Asia (Moonlight et al. 2018), of which 4 caulescent species are distributed in Himalayan areas (Doorenbos et al. 1998), i.e. *Begonia goniotis* C.B. Clarke (Hooker, 1879), *B. griffithiana* (A.DC.) Warb. (Warburg 1894; basionym: de Candolle 1859), *B. nepalensis* (A.DC.) Warb. (Warburg 1894; basionym: de Candolle 1859) and *B. sandalifolia* C.B. Clarke (Hooker, 1879).

## Taxonomy

### Begonia medogensis

Taxon classificationPlantaeCucurbitalesBegoniaceae

JianW.Li, Y.H.Tan & X.H.Jin sp. nov.

urn:lsid:ipni.org:names:77186060-1

Figure 1

#### Diagnosis.

*Begonia medogensis* is morphologically similar to *B. goniotis*, *B. griffithiana*, *B. nepalensis* and *B. sandalifolia*, but can be easily distinguished from them by having leaves ovate-lanceolate, 6.0–8.0 × 1.5–2.5 mm, base slightly asymmetric, margins remotely and irregularly denticulate; triangular to lanceolate stipules; staminate flowers with outer 2 segments broadly ovate, inner 2 spathulate; pistillate flowers with perianth segments unequal, outer 4 larger, broadly ovate, inner 1 smallest, spathulate; cylindroid ovary, larger wing oblong, apex truncate.

#### Type.

CHINA. Tibet, Medog County, Beibeng town, semi-evergreen forest in a subtropical area, 29°15'09"N, 95°13'31"E. 1381 m a.s.l., 16 November 2017, flowering, *Xiaohua Jin, Jianwu Li, Xilong Wang & Chengwang Wang 19331* (holotype: HITBC!, isotype: HITBC!, PE!, K!)

**Figure 1.**
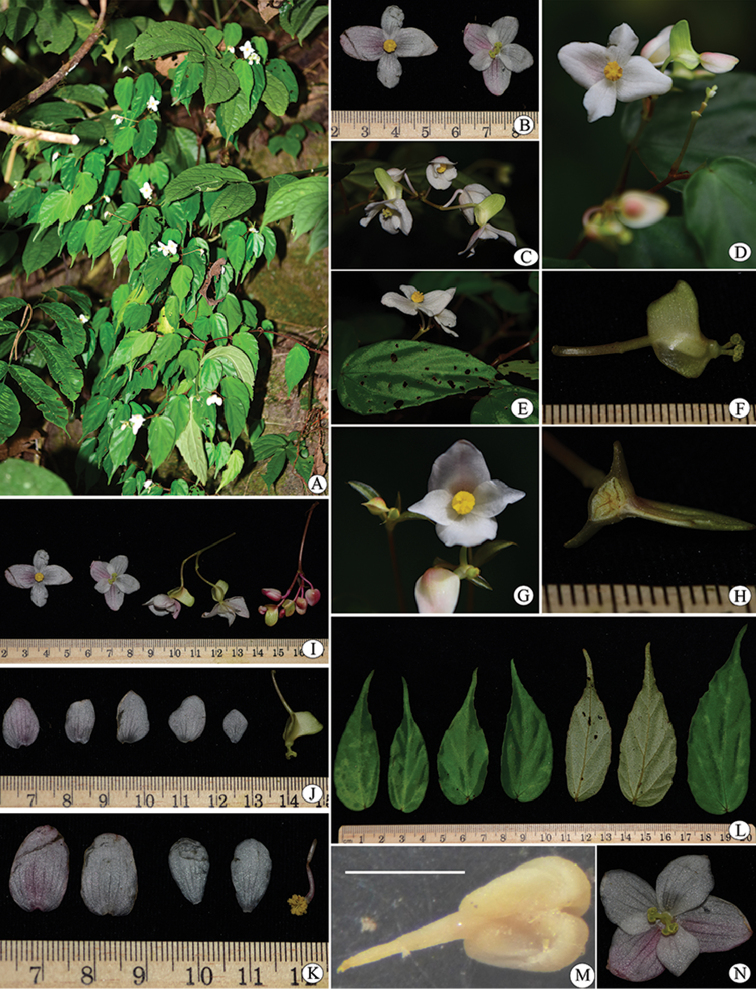
*Begonia medogensis* JianW.Li, Y.H.Tan & X.H.Jin. (photographed by Jian-Wu Li). **A** Habitat **B–E** Flowers **F** Pedicel and ovary (showing large wing) **G** Male flowers (face view) **H** Ovary (showing loculus) **I** Flowers **J** Dissection of female flower **K** Dissection of male flower **L** Leaves **M** Anther with filament (under dissection mirror, bar = 1 mm) **N** Female flower (face view).

#### Description.

Perennial herbs, caulescent, erect. Rhizomes short, stout. Stems reddish-purple, densely pubescent, 0.3–1.0 m tall, with internode 6–15 cm long, upper part well-branched, with internode 2–5 cm long. Leaves cauline; stipules persistent, triangular to lanceolate, 6.0–8.0 × 1.5–2.5 mm, apex acuminate to cuspidate; petiole 1.2–2.7 cm long, densely pubescent; leaf blade ovate-lanceolate, slightly asymmetric, 6.0–10.0 × 2.0–3.7 cm, adaxially green, slightly hairy, abaxially greenish-white, hairy on venation, venation palmate-pinnate, 5–8-veined, base slightly oblique, rounded to subcordate, margins remotely and irregularly denticulate, apex caudate-acuminate. Inflorescences terminal and axillary, sub-corymb, monoecious, to 6 cm, sub-pendulous, peduncles 2.7–4.0 cm long, secondary 0.5–1.0 cm long, terminally with 1–5 flowers; floral bracts lanceolate to ovate-lanceolate, 5.0–10.0 × 1.7–5.0 mm, thickly papery, glabrous, apex acuminate. Staminate flowers: pedicel 10–15 mm long; tepals 4, white, outer 2 broadly ovate, 15–17 × 12–14 mm, tinted with pink, glabrous, apex rounded; inner 2 spathulate, 11–14 × 6–8 mm, glabrous, apex obtuse to rounded; stamens 60–80; filaments free, 0.5–1.2 mm long, sub-equal, fused at base into a column; anthers broadly lorate, 0.8–1.1 mm long, apex emarginate. Pistillate flowers: pedicel 11–20 mm long, tepals 5, white, unequal, glabrous, outer 4 broadly ovate, the outmost 2 tinted with pink, 12–14 × 9–12 mm, apex rounded, other 2 slightly larger, 13–15 × 10–12 mm, apex obtuse to rounded; the inner 1 smallest, spathulate, 8–10 × 7–9 mm, apex rounded; ovary glabrous, 2-loculed; placentae axile, bilamellate; styles 2, fused at base; stigmas 2-cleft, spiralled. Capsule sub-pendulous, cylindroid, 5.0–8.0 × 1.5–2.5 mm; wings 3, adaxial 1 larger, oblong, 7–9 mm broad, apex truncate, lateral 2 less developed, 2–3 mm broad.

#### Phenology.

Flowering from October to December.

#### Distribution and habitat.

This new species grows in subtropical areas in Beibeng town, Medog County, Tibet, China, at an elevation of 700–1400 m and in Putao district, Kachin state, Myanmar, at an elevation of 600–1200 m.

#### Etymology.

The species is named after the holotype locality, Medog County, in Tibet, China.

#### Additional specimens examined

**(paratype).** MYANMAR. Putao district, Kachin state, in tropical montane forest, 27°37'15"N, 97°53'14"E. 900 m a.s.l., 1 December 2016, flowering, *Myanmar Exped. M0727* (HITBC!).

#### Note.

Morphologically, the new species is similar to *B. goniotis*, *B. griffithiana*, *B. nepalensis* and *B. sandalifolia*, but differs from them by the shape of stipules and leaves, base and margins of leaves, both male and female flowers having unequal perianth segments, cylindroid ovary etc. (see Table 1).

**Table 1.** Differences between *Begonia medogensis*, *B. goniotis*, *B. griffithiana*, *B. nepalensis* and *B. sandalifolia*.

Character	*B. medogensis*	*B. goniotis*	*B. griffithiana*	*B. nepalensis*	*B. sandalifolia*
Stem	internode 6–15 cm long	internode 4–10 cm long	internode 3–9 cm long	internode 4–11 cm long	internode 9–20 cm long
Stipules	persistent, triangular to lanceolate, 6.0–8.0 × 1.5–2.5 mm	persistent, ovate, 6–13 × 4–10 mm	persistent, lanceolate, 4–13 × 1–2 mm	deciduous, lanceolate	persistent, oblong, 6–10 × 2–4 mm
Leaves	petiole 1.2–1.7 cm long, ovate-lanceolate, base slightly asymmetric, 6.0–10.0 × 2.0–3.7 cm, margins remotely and irregularly denticulate, apex caudate-acuminate	petiole 1.0–4.6 cm long, lanceolate, base strongly asymmetric, 10–15 × 0.6–2.5 cm, margins entire or serrate near apex, apex long acuminate	petiole 0.2–1 cm long, oblong-lanceolate to lanceolate, base strongly asymmetric, 5–18 × 2–5 cm, margins serrulate or with small teeth at ends of the main veins, apex acuminate	petiole 1–3 cm long, ovate-lanceolate, base strongly asymmetric, 15–17 × 4–11 cm, margins shallowly dentate, apex caudate-acuminate	petiole 0.7–1.3 cm long, ovate to oblong, base strongly asymmetric, 10–15 × 3–5 cm, margins entire or serrate, apex acuminate
Male flower	tepals 4, outer 2 broadly ovate, 15–17 × 12–14 mm, tinted with pink, inner 2 spathulate, 11–14 × 6–8 mm, white, stamens 60–80, anthers broadly lorate	not seen	tepals 4, outer 2 orbicular to oblong, 4–14 × 2–11 mm, pale pink to white, inner 2 lanceolate to linear, 3–9 × 1–4 mm, pale pink, stamens 20–30, anthers elliptic-globose	tepals 2, ovate, 7–10 × 7–9 mm, pale pink to white, stamens 20–40, anthers elliptic globose	tepals 4, outer 2 round, 18 × 18 mm, inner 2 oblong, stamens ca. 50, anthers obovoid
Female flower	tepals 5, unequal, outer 4 broadly ovate, the outmost 2 tinted with pink, 12–14 × 9–12 mm, other 2 slightly larger, 13–15 × 10–12 mm; the inner 1 smallest, spathulate, 8–10 × 7–9 mm	not seen	tepals 5–6, equal, 3–4 larger and 1–2 smaller, oblong elliptic to obovate orbicular, outer tepals 6–11 × 5–7 mm, pale pink to white, inner tepals smaller	tepals 4–5, equal, outer tepals 10–15 × 7–10 mm, pale pink to white, inner tepals smaller	not seen
Ovary	cylindroid, 5–8 × 1.5–2.5 mm, larger wing oblong, 7–9 mm broad, apex truncate	ellipsoid, 10–13 × 6 mm, larger wing oblong, 10–16 mm broad, apex rounded	oblong-ellipsoid, 5–7 × 2–3 mm, larger wing triangular, 12–18 mm broad, apex obtuse	narrowly ellipsoid, 12–15 × 5 mm, larger wing oblong, 15–23 mm broad, apex truncate	ellipsoid, 13–16 × 6 mm, larger wing oblong, 13–16 mm broad, apex rounded

## Supplementary Material

XML Treatment for Begonia medogensis
